# Blood-based Alzheimer's disease biomarkers and cognitive decline in essential tremor

**DOI:** 10.1177/13872877251404035

**Published:** 2025-12-12

**Authors:** Tomer O Guy, Ali Ghanem, Diane S Berry, Nora C Hernandez, Vibhash D Sharma, Silvia Chapman, Stephanie Cosentino, Elan D Louis

**Affiliations:** 1 Department of Neurology, University of Texas Southwestern Medical Center, Dallas, TX, USA; 2 Department of Neurology, Henry Ford Health System, Detroit, MI, USA; 3 Peter O’Donnell Jr. Brain Institute, University of Texas Southwestern Medical Center, Dallas, TX, USA; 4 Taub Institute for Research on Alzheimer's Disease and the Aging Brain, Columbia University Irving Medical Center, New York, NY, USA; 5 Gertrude H. Sergievsky Center, Columbia University Irving Medical Center, New York, NY, USA; 6 Department of Neurology, Columbia University Irving Medical Center, New York, NY, USA

**Keywords:** Alzheimer's disease, biomarkers, blood-based biomarkers, cognition, dementia, essential tremor

## Abstract

**Background:**

Two epidemiological studies demonstrated an association between essential tremor (ET) and prevalent dementia as well as substantially elevated risks of incident dementia among ET cases. At this early point, the underlying pathophysiology of ET-dementia is not known. In vivo biomarkers of Alzheimer's disease (AD) and neurodegeneration could help bridge the gap between the pathophysiological processes that present in the context of ET-dementia.

**Objective:**

Examine blood concentrations of t-tau, p-tau181, p-tau217, Aβ_42/40_, neurofilament light (NfL) and glial fibrillary acidic protein (GFAP) in ET with a range of cognitive diagnoses.

**Methods:**

40 ET cases (mean age = 81.5 ± 7.3; including 20 normal cognition (NC), 12 cognitively normal with some weaknesses, 4 mild cognitive impairment, and 4 dementia) were enrolled in a study of cognitive performance in ET, during which phlebotomy was performed.

**Results:**

Greater cognitive difficulty was associated with higher blood concentrations of p-tau217, p-tau181, GFAP, and NfL, and a lower Aβ_42/40_ ratio (*p* tests for trend < 0.05). Cases with dementia had marginally higher concentrations of p-tau217 (*p* = 0.06) and higher concentrations of GFAP and NfL (*p* < 0.05) than cases with NC. Furthermore, higher concentrations of p-tau217, GFAP, and NfL were associated with lower cognitive test scores across multiple cognitive domains (*p* < 0.05).

**Conclusions:**

Albeit based on a small sample of cases, our findings suggest a potential role of blood-based biomarkers as markers for cognitive function in ET patients. Cognitive decline in ET may be due to underlying neurodegenerative processes involving tau and perhaps Aβ pathology.

## Introduction

Essential tremor (ET) is one of the most common movement disorders, estimated to affect 7 million people in the United States.^1,2^ The pathophysiology of ET has not been fully elucidated although there is considerable postmortem evidence that it is neurodegenerative, with postmortem changes observed primarily in the cerebellar cortex.^3–7^

Independent studies from around the world have shown that ET cases have poorer cognitive performance than age-matched non-ET controls.^8^ In addition to studies that implicate a range of cognitive deficits in ET, there is an emerging understanding that cognitive problems in ET can be progressive^9^ and that the rate of progression seems to be above and beyond that expected in controls.^9^ Two prospective, population-based, epidemiological studies, one in Madrid and the other in New York, have demonstrated an association between ET and prevalent dementia.^10,11^ Furthermore, in both studies, the risk of incident dementia was higher among individuals with baseline ET than among individuals without baseline ET (relative risk [RR] = 1.–1.89).^10,11^ One additional study found that a subset of ET cases (i.e., those with older onset/shorter duration) had increased risk for dementia.^12^ Moreover, recently completed studies estimate that the rate of conversion from normal cognition (NC) to dementia in ET is approximately three times higher than in the non-ET population.^13,14^

Despite the observed increases in odds and risk of dementia in individuals with ET, the underlying pathophysiology of ET-dementia is not known. There are several possibilities. First, several studies indicate that the brains of ET patients exhibit an increase in tau pathology^15,16^ suggesting that individuals with ET patients are prone to developing a tauopathy.^15,16^ In the first of these studies, Alzheimer's disease (AD) Braak stage, which reflects spread of neurofibrillary tau tangles, was assessed in 40 dementia-free ET cases and 32 non-ET controls.^15^ The authors reported that Braak AD stage was higher in ET than controls (1.9 ± 1.3 versus 0.8 ± 1.0, p˂0.001), indicating a possible predisposition in ET to accumulating tau pathology. By contrast, in that study, neuritic plaque counts were similar in both groups (0.6 ± 0.9 in ET versus 0.5 ± 0.6 controls, p = 0.83).^15^ In a second study, 26 ET cases were compared with 73 controls; all had had detailed longitudinal neuropsychological testing.^16^ The investigators reported that ET cases with both NC and mild cognitive impairment (MCI) had higher neurofibrillary tangle counts than controls with these cognitive diagnoses, further suggesting an increased susceptibility to tau pathology among ET cases.^16^ A recent study used cytometry-based tau biosensor assays in brain homogenates of ET cases with tau pathology to investigate tau assembly conformation. This method quantifies tau seeding activity by detecting the aggregation of tau-fluorescent proteins from soluble state within biosensor cells.^17^ The authors noted that in a pathologically confined subset of eight ET cases with significant tau pathology, tau assembly cores were identical to those seen in AD and primary age-related tauopathy (PART).^17^ Although on a molecular level, these results suggested that the underlying pathological change in ET-dementia is AD and PART, that study was limited to only eight ET cases and studies of additional cases are needed.^17^

A second possibility is that Lewy pathology underlies the association between ET and dementia. Several cohort studies have demonstrated that patients with ET have a propensity for Lewy body disease,^10,18^ and postmortem studies have demonstrated that approximately one-in-four ET cases have Lewy pathology on postmortem brain examination.^19,20^ This raises the question as to whether a significant proportion of the dementia in ET could be linked to the underlying presence of Lewy body disease.

A third possibility is that the dementia in ET is the result of vascular pathology. White matter hyperintensity burden has been reported to be elevated in ET in one study,^21^ and was linked with cognition in ET in another study.^22^

Given these competing hypotheses, studies that further shed light on the underlying causes for dementia in ET are of value, and the current analyses were designed to further explore the hypothesis that AD could underlie this dementia.

The AD field has undergone a revolutionary change in the past several years with the advent and validation of blood-based biomarkers (BBB) for AD.^23,24^ Specifically, the 2024 Revised Alzheimer's Association criteria for diagnosing and staging AD identify plasma analytes of core AD pathologies (amyloid-β [Aβ] and tau) as acceptable in vivo biomarkers for AD diagnosis.^25^ In particular, measuring the ratio of Aβ_42_ concentrations to Aβ_40_ concentrations (Aβ_42/40_), in conjunction with concentrations of phosphorylated tau 217 (p-tau217) in blood plasma is now considered equivalent to diagnosis of AD by cerebrospinal fluid.^23,24^^26–29^ In addition, the revised diagnostic criteria highlight the utility of neurofilament light (NfL; a measure of large caliber axonal injury) and glial fibrillary acidic protein (GFAP; a measure of astrocytic activation) for measuring neurodegeneration in AD and related diseases (e.g., Parkinson's disease [PD] and Lewy body dementia).^23,24,30,31^ Examination of blood concentrations of p-tau217, p-tau181, total tau, Aβ_42/40_, NfL, and GFAP will thus allow examination of the pathophysiological processes that are present in the context of ET-dementia.

In this study, we hypothesized that if AD were responsible for the dementia in ET, then blood concentrations of the above-mentioned BBB of AD would track with cognitive diagnosis and cognitive performance in ET. That is, that AD biomarkers would relate to dementia in ET. Therefore, we (1) present data on blood concentrations of each of these biomarkers in 40 ET patients who presented with a range of cognitive diagnoses (NC, MCI, and dementia), and (2) explore the associations between these biomarker concentrations and cognitive performance across these cases.

## Methods

### Parent study and current sample of participants

Participants were enrolled in an ongoing, prospective study of cognitive performance (Clinical Pathological Study of Cognitive Impairment in Essential Tremor, NINDS R01 NS086736). The study was approved by Yale University, Columbia University, and the University of Texas Southwestern Medical Center. Nationwide enrollment began in July 2014, and cases to date reside in 43 US states. Eligibility requirements are (1) a diagnosis of ET; (2) a baseline age of at least 55 years; (3) no history of brain surgery as a treatment for ET at baseline; and (4) enrollment as an eventual donor in the Essential Tremor Centralized Brain Repository. All participants signed a written consent form. A sub-study aimed to evaluate BBB in 40 ET patients, evaluating the study subjects who were consecutively enrolled between February and November 2023.

### Study evaluation

*Overview.* Participants included in the parent and the sub-study underwent an extensive in-person home evaluation conducted by trained research assistants. The evaluation included clinical questionnaires, a videotaped neurological examination, and an extensive neuropsychological test battery. Included in these questionnaires were the total number of prescription medications, the Cumulative Illness Rating Scale score (CIRS; 14 items, range = 0–42 [maximum]),^32^ and the Geriatric Depression Scale score (GDS; 30 items, range = 0 [no depressive symptoms] – 30 [severe depressive symptoms]).^33^

*Confirmation of ET diagnosis.* An experienced movement disorders neurologist examined the videotaped neurological examination, assigned a total tremor score (a measure of the severity of action tremor, range = 0–36 [most severe])^34^ and confirmed the clinical diagnosis of ET based on the Washington Heights Inwood Genetic Study of ET criteria, which are both reliable and valid.^35–37^

*Neuropsychological test battery.* As described in detail, all participants underwent an extensive cognitive test battery with little demands on motor functioning.^38,39^ The Montreal Cognitive Assessment (MoCA),^40^ a 30-point validated and sensitive screening test for assessing mild cognitive impairment and dementia, was administered during the cognitive assessment. We also used a wide range of neuropsychological tests to evaluate the following cognitive domains: For attention, we used the Wechsler Adult Intelligence Scale IV (WAIS-IV) Digit Span Forward Test^41^ and the Oral Symbol-Digit Modalities Test.^42^ For language, we used the 30-item Boston Naming Test.^43^ The visuospatial domain was evaluated via the Benton Judgement of Line Orientation,^44^ Benton Facial Recognition test,^45^ and the WAIS-IV Visual Puzzles Test.^41^ For memory, we used the California Verbal Learning Test II,^46^ the Wechsler Memory Scale-Revised: Logical Memory,^47^ and the Wechsler Memory Scale IV: Verbal Paired Associates Test.^47^ Executive function was tested using the Delis- Kaplan Executive Function System (D-KEFS) Sorting Test, the D-KEFS Verbal Fluency Test, the D-KEFS Color Word Interference Test, the D-KEFS Twenty Questions Test,^48^ and the Wechsler Adult Intelligence Scale IV (WAIS-IV) Digit Span Backward Test.^41^

*Cognitive diagnosis.* For the purposes of diagnosis, all raw neuropsychological tests were converted into standardized scores using publicly available, clinical normative data provided by test publishers. Standardized scores equal to or less than −1.5 standard deviations below the mean were defined as ‘impaired’. As described in detail,^38,39^ a neuropsychologist and geriatric psychiatrist then reviewed each participant's neuropsychological performance, in conjunction with information from mood inventories and the Clinical Dementia Rating (CDR)^49^ scale, during a diagnostic consensus conference to determine a cognitive diagnosis. Specifically, normal cognition (NC) was assigned for patients who had no functional impairment (CDR = 0) and performed within normal limits on all neuropsychological tests (z > −1.5). An MCI diagnosis was assigned to any case with a CDR of 0.5 and impairment (z-score ≤ −1.5) on two MCI-designated tests. Specific “criterion” tests within each domain were selected to operationally define impairment for the diagnosis of MCI. Criterion measures were selected based on: 1) the relative purity of measurement for the construct under evaluation (e.g., Judgment of Line Orientation^44^ for the spatial domain given its relatively lesser demand on executive functioning than Visual Puzzles)^50^; 2) the demonstrated utility of measures in previous studies; and 3) the general availability of the measure to researchers who may wish to replicate the current findings. Two tests per domain were used based on the recommendation of the Movement Disorder Society Task Force on PD-MCI.^51^ The domain of executive functioning was an exception, wherein we evaluated four different scores of executive functioning given the heterogeneous nature of this domain and its known role in ET. In order to meet criteria for single domain impairment, impairment was required on at least 50% of the tests in that domain (i.e., 1 of 2 the two memory criterion tests, and 2 of the 4 executive functioning criterion tests). The selected tests were as follows: CVLT-II long-delay,^46^ and the Logical Memory delay^52^ (**memory)**, Digit Span Forward^50^ total score and Symbol Digit Modalities Test^42^ total oral score (**attention)**, Verbal Fluency letter fluency score, Color-Word Interference inhibition score, Sorting Test free sorting description score, and Twenty Questions total weighted achievement score^53^ (**executive function)**, Judgment of Line Orientation^44^ total score and Benton Facial Recognition total score^45^ (**visuospatial ability)**, Boston Naming Test^54^ total score and Token Test^44^ total score (**language)**.

Individuals who scored below normal limits on one or two neuropsychological tests (not including the Mini-Mental State Examination or MoCA), but who did not meet criteria for MCI were labeled as “cognitively normal with some weaknesses” (CN + SW). This diagnostic category distinguished individuals whose cognition is entirely within normal limits from those with some variability in cognitive testing which may or may not be of clinical significance. Dementia was assigned if the CDR was 1 or higher, with impairments in multiple domains.

### Sample collection, storage, and blood biomarker measurement

After written informed consent was obtained, phlebotomy was performed by a trained research assistant, and 10 mL of blood was collected in an EDTA-containing tube labeled using a de-identified code. Samples were then centrifuged to obtain the plasma and immediately stored frozen at −80°C.^55^ Before processing, all samples were thawed and sent to the Microarray Core BioCenter at the University of Texas Southwestern Medical Center, Dallas, TX, USA, for duplicate analysis using the Simoa^©^ platform (Quanterix^©^, Billerica, MA, USA) using the following kits: Human Neurology 3-Plex A assay (N3PA) (for t-tau), human p-tau-181 Advantage V2 assay (for p-tau181), ALZpath assay (for p-tau217), NF-Light V2 Advantage (for NfL), and Human Neurology-Plex E (N4PE) assay (for Aβ_40_ and Aβ_42_, and GFAP). Analyses were carried out without knowledge of clinical information or diagnosis. Reported values (pg/mL) represent the mean of two readings per sample.

### Statistical analysis

In order to create cognitive domain outcome scores, we converted the raw scores of each test to z-scores based on *within-sample normative* data (i.e., mean and standard deviation of baseline scores among all ET cases diagnosed as cognitively normal) following established protocols.^38,56^ We then constructed *cognitive domain scores* at each evaluation by averaging the z*-*scores of tests included in a given domain. A global performance z-score was created by calculating the mean of the five domain scores. Z-scores were not demographically adjusted, as statistical analyses considered the potential confounding effects of age, education, sex and several other covariates.

Distributions of demographic and clinical variables for the sample appear in Table 1. We examined the normality of test variables using a Kolmogorov-Smirnov Test and used nonparametric tests as needed. We tested differences in biomarker concentrations across cognitive diagnosis categories using a Jonckheere-Terpstra test for trend (Table 1). Furthermore, using a Mann-Whitney test, we directly compared ET cases with dementia to those with NC (Table 1). Finally, a series of stepwise regression analyses was conducted in which each individual biomarker concentration was included with age, sex, years of education, depressive symptoms (GDS), cumulative illness (CIRS), and number of medications as explanatory variables, and the ordinal measure of cognitive diagnosis (i.e., 1 = NC, 2 = CN + SW, 3 = MCI, 4 = dementia) served as the outcome (Table 2). Parallel series of stepwise regression equations were computed in which each biomarker concentration along with age, sex, education, GDS, CIRS, and number of medications served as potential predictors of outcomes on the MoCA and on z-scores derived from each cognitive domain (Table 2).

**Table 1.** Demographic, clinical, and biomarker characteristics of 40 ET cases by cognitive diagnosis.

	Total	NC	CN ± SW	MCI	Dementia	*p* (J-T)	*p* (M-W)
Variable							
N	40	20	12	4	4		
Age (y)^a^	80.8 ± 8.9	78.2 ± 6.1	78.4 ± 9.7	87.8 ± 6.9	93.5 ± 6.5	**0**.**002**	**0**.**001**
Female sex^b^	24 (60.0)	13 (65)	7 (58)	1 (25)	3 (75)	0.45^d^	0.70 ^d^
Education (y)^a^	16.0 ± 2.6	16.6 ± 2.2	15.2 ± 2.9	16.5 ± 1.0	14.5 ± 3.8	0.12	0.21
Age of tremor onset (y)^a^	39.9 ± 22.0	43.8 ± 22.2	33.1 ± 20.5	37.3 ± 26.2	43.5 ± 8.4	0.36	0.91
Tremor duration (y)^a^	40.9 ± 20.8	34.5 ± 19.2	45.3 ± 17.8	50.5 ± 24.3	50.0 ± 30.1	**0**.**05**	0.31
Tremor severity (TTS)^a^	22.2 ± 5.4	22.9 ± 5	21.2 ± 4.8	23.3 ± 8.1	20.8 ± 7.5	0.42	0.56
Family history of ET^a^	21 (52.5)	10 (50)	7 (58)	2 (50)	2 (50)	0.93	0.50
Number of prescription medications^a^	5.0 ± 3.3	4.0 ± 3.1	4.9 ± 3.0	8.3 ± 1.5	6.3 ± 4.7	**0**.**03**	0.36
CIRS^a^	7.2 ± 3.3	6.6 ± 3.5	7.7 ± 3.14	8.0 ± 0.6	8.3 ± 4.3	0.21	0.53
GDS^a^	5.5 ± 4.7	5.5 ± 5.3	4.6 ± 3.9	6.3 ± 3.9	7.8 ± 5.1	0.44	0.35
Biomarker^c^							
t-tau	3.2; 3.4 ± 1.2; 5.3	3.4; 3.5 ± 1.2; 4.0	3.2; 3.2 ± 0.8; 2.4	*	3.4; 4.1 ± 2.3; 4.5	0.44	0.90
p-tau181	29.7; 30.7 ± 16.6; 88.3	23.1; 24.6 ± 6.4; 18.5	30.8; 31.2 ± 5.7; 17.3	*	31.5; 52.1 ± 44.3; 81.1	**0**.**01**	0.24
p-tau217	0.4; 0.5 ± 0.3; 1.4	0.3; 0.4 ± 0.2; 0.6	0.6; 0.7 ± 0.3; 0.9	0.6; 0.8 ± 0.6; 1.26	0.5; 0.7 ± 0.5; 1.2	**0**.**002**	0.06
Aβ_42/40_	0.05; 0.05 ± 0.02; 0.12	0.06; 0.06 ± 0.03; 0.12	0.05; 0.05 ± 0.01; 0.03	0.05; 0.05 ± 0.005; 0.01	0.05; 0.05 ± 0.02; 0.04	**0**.**007**	0.12
GFAP	150.7; 162.5 ± 68.7; 346.9	111.6; 117.7 ± 35.9; 123.7	194.2; 196.0 ± 45.9; 151.8	148.7; 168.1 ± 43.4; 90.5	192.9; 232.8 ± 127.5; 282.04	**0**.**001**	**0**.**02**
NfL	22.9; 26.9 ± 12.2; 63.2	21.5; 22.8 ± 8.2; 30.0	22.0; 23.2 ± 7.3; 26.3	36.2; 37.0 ± 9.1; 18.3	44.6; 48.3 ± 17.7; 40.1	**0**.**003**	**0**.**002**
MoCA	25.0 ± 3.4	26.6 ± 1.9	26.2 ± 1.6	20.8 ± 1.9	18.3 ± 3.5	**0**.**001**	**0**.**001**
Cognitive Z Scores ^a^							
Attention	−0.3 ± 0.9	0.1 ± 0.7	−0.4 ± 0.7	−1.8 ± 0.6	−1.4 ± 0.5	**0**.**001**	**0**.**002**
Executive Function	0.1 ± 0.8	0.6 ± 0.4	0.06 ± 0.6	−1.3 ± 0.7	−1.2 ± 0.5	**0**.**001**	**0**.**001**
Language	0.6 ± 1.2	1.1 ± 0.4	0.6 ± 1.0	−1.2 ± 1.9	−0.2 ± 1.9	**0**.**007**	0.23
Memory	0.05 ± 1.2	0.8 ± 0.6	−0.06 ± 0.9	−1.1 ± 0.1	−2.3 ± 0.2	**0**.**001**	**0**.**001**
Visuospatial	0.03 ± 0.8	0.3 ± 0.7	0.06 ± 0.6	−1.1 ± 0.5	−0.7 ± 0.6	**0**.**002**	**0**.**04**
Global	0.02 ± 0.9	0.6 ± 0.4	0.06 ± 0.3	−1.3 ± 0.7	−0.5 ± 0.8	**0**.**001**	**0**.**001**

^a^ Values represent mean ± standard deviation.

^b^ Values represent number (percent).

^c^ Values represent mean biomarker concentrations (pg/mL), median ± standard deviation, range.

^d^ Chi-Square test.

J-T: Jonckheere-Terpstra test; M-W: Mann-Whitney test. Bolded values are significant at *p* < 0.05. *Assay was added later than that of NfL, GFAP, and Aβ_42/40_; hence we have fewer data points.

Aβ: amyloid-β; CIRS: Cumulative Illness Rating Scale score; CN + SW: cognitively normal with some weaknesses; GDS: Geriatric Depression Scale; GFAP: glial fibrillary acidic protein; MCI: mild cognitive impairment; MoCA: Montreal Cognitive Assessment; NC: normal cognition; NfL: neurofilament light chain; p-tau181: phosphorylated tau 181; p-tau217: phosphorylated tau 217; t-tau: total tau.

**Table 2.** Final equation associations between individual biomarkers and cognitive performance measures: stepwise regression analyses.

Biomarker	Measure of Cognitive Performance		
(Predictor)	(Outcome)	Beta	*p*
t-tau	Cognitive Diagnosis	n/a	n/a
	MoCA	n/a	n/a
	Attention z-score	n/a	n/a
	Executive Function z-score	n/a	n/a
	Language z-score	n/a	n/a
	Memory z-score	n/a	n/a
	Visuospatial z-score	n/a	n/a
	Global z-score	n/a	n/a
p-tau181	Cognitive Diagnosis	0.60	**0.004**
	MoCA	−0.76	**0.001**
	Attention z-score	n/a	n/a
	Executive Function z-score	n/a	n/a
	Language z-score	−0.54	**0.01**
	Memory z-score	n/a	n/a
	Visuospatial z-score	n/a	n/a
	Global z-score	−0.56	**0.002**
p-tau217	Cognitive Diagnosis	n/a	n/a
	MoCA	n/a	n/a
	Attention z-score	n/a	n/a
	Executive Function z-score	n/a	n/a
	Language z-score	−0.60	**0.001**
	Memory z-score	n/a	n/a
	Visuospatial z-score	n/a	n/a
	Global z-score	−0.38	**0.009**
Aβ_42/40_	Cognitive Diagnosis	−0.29	**0.048**
	MoCA	n/a	n/a
	Attention z-score	n/a	n/a
	Executive Function z-score	0.32	**0.03**
	Language z-score	n/a	n/a
	Memory z-score	n/a	n/a
	Visuospatial z-score	n/a	n/a
	Global z-score	n/a	n/a
GFAP	Cognitive Diagnosis	0.39	**0.02**
	MoCA	n/a	n/a
	Attention z-score	−0.37	**0.03**
	Executive Function z-score	n/a	n/a
	Language z-score	−0.37	**0.04**
	Memory z-score	n/a	n/a
	Visuospatial z-score	n/a	n/a
	Global z-score	−0.44	**0.003**
NfL	Cognitive Diagnosis	0.64	**0.001**
	MoCA	−0.82	**0.001**
	Attention z-score	n/a	n/a
	Executive Function z-score	−0.33	
	Language z-score	−0.48	**0.005**
	Memory z-score	−0.46	**0.006**
	Visuospatial z-score	−0.53	**0.002**
	Global z-score	−0.56	**0.001**

Standardized betas and associated *p* values between a given biomarker and cognitive performance measure are from final equations for stepwise multiple regression analyses predicting cognitive performance from biomarker, age, sex, education, GDS (Geriatric Depression Scale), CIRS (Cumulative Illness Rating Scale), and number of medications. *p* values ≤ 0.05 appear in bold. N/a = beta and alpha are not reported as biomarker was not included in final equation predicting specific measure of cognitive performance.

Cognitive diagnosis = ordinal scale in which 1 = normal cognition, 2 = cognitively normal with some weaknesses, 3 = mild cognitive impairment, 4 = dementia. Higher cognitive diagnosis values indicate poorer cognitive functioning. Higher values on all other cognitive measures indicate better cognitive functioning.

Aβ: amyloid-β; GFAP: glial fibrillary acidic protein; MoCA: Montreal Cognitive Assessment; NfL: neurofilament light chain; p-tau181: phosphorylated tau 181; p-tau217: phosphorylated tau 217; t-tau: total tau.

## Results

### Subject characteristics

There were 40 ET cases. Their mean age was 80.8 ± 8.9 years, and mean tremor duration was 40.9 ± 20.8 years (Table 1).

### Blood biomarker concentrations by cognitive diagnosis

The blood concentration of each biomarker was determined for ET patients with each cognitive diagnosis (NC, CN + SW, MCI and dementia, Table 1). In tests for trend, greater cognitive difficulty was associated with higher blood concentrations of p-tau181, p-tau217, GFAP, and NfL and a lower Aβ_42/40_ ratio (Table 1). We also directly compared ET cases with dementia to those with NC. Cases with dementia had marginally higher concentrations of p-tau 217 (*p* = 0.06) and higher concentrations of GFAP and NfL (*p* values = 0.02 and 0.002, respectively) than cases with NC (Table 1).

A series of stepwise multiple regression analyses in which an individual biomarker concentration and age, sex, education, GDS, CIRS, and number of medications were entered as potential explanatory variables, and the ordinal measure of cognitive diagnosis (i.e., 1 = NC, 2 = CN + SW, 3 = MCI, 4 = dementia) served as the outcome (Table 2) revealed a similar pattern of associations, although p-tau217 no longer predicted cognitive diagnosis (Table 2).

### Blood biomarker concentrations by MoCA and by cognitive domain scores

A series of parallel stepwise regression equations was also computed in which each biomarker concentration as well as age, sex, education, GDS, CIRS, and number of medications served as potential predictors of performance on the MoCA, or on the z-scores computed for individual cognitive domains (Table 2). These revealed that higher concentrations of NfL were associated with lower cognitive performance scores across multiple domains (Table 2). Poorer performance in selected cognitive domains was also revealed for cases with higher levels of p-tau181, p-tau217, and GFAP, and lower Aβ_42/40_ ratios (Table 2).

## Discussion

Despite the observed increases in odds and risk of dementia in individuals with ET, the underlying pathophysiology of ET-dementia is not known. There are several possibilities, and the current analyses were designed to further explore the hypothesis that AD could underlie this dementia. The results of this study support this hypothesis, although the modest sample size should be noted. More specifically, several associations indicated that AD biomarkers were related to cognitive impairment and dementia in this sample of ET cases. As such, blood p-tau217 concentration (1) rose across the cognitive diagnoses, (2) was marginally higher in dementia than in NC, and (3) was moderately associated with cognitive performance across several cognitive domains. In addition, p-tau181 rose across the cognitive diagnoses and Aβ_42/40_ declined across cognitive diagnosis to some extent, although neither differentiated demented from NC, and their association with cognitive performance across cognitive domains was less robust. Prior studies have demonstrated that p-tau217 can differentiate between AD and other neurodegenerative diseases and has high accuracy when identifying different stages of AD and Aβ pathology longitudinally.^57,58^ In addition, p-tau217 has been shown to outperform p-tau181 in discerning between AD and other diseases such as frontotemporal lobar degeneration syndromes.^57,59^

In this study, demented cases had higher NfL concentrations than cases with MCI, NC + SW, and NC, and NfL concentrations were highly correlated with worse cognitive performance. These results expand on our previous knowledge on blood NfL concentrations in ET. Previously, we reported that ET patients had significantly higher NfL concentrations than healthy controls.^60,61^ Our results also align with prior studies of GFAP, another biomarker of neurodegeneration in AD, shown to be negatively correlated with the Mini-Mental State Examination score and cognitive function in multiple domains.^62^

One question is how the values we observed for blood concentrations of p-tau181, p-tau217, and Aβ_42/40_ in our 4 demented ET cases compare with those of individuals without ET who have AD. The Alzheimer's Disease Center Fluid Biomarker (ADCFB) Initiative provides the following diagnostic cut off values for AD: 0.47 pg/mL (for p-tau217) and 4.09 pg/mL (for p-tau181). Three (75.0%) of our four ET cases had a p-tau217 value at or above this cut off value and three (100%) of our three ET cases with available data had a p-tau181 value above this cut off value. Furthermore, three (75%) of our four ET cases had an Aβ_42/40_ value below the published cut off value of 0.58 pg/mL. Hence, the majority of cases in our sample had values that fell in the published AD range.^63^

An additional question is how the values we observed for blood concentrations of these biomarkers in our 20 ET cases with NC compare with those of individuals without ET who have NC. A limitation of our study is that we did not collect data from individuals without ET. Nonetheless, relevant published data from neurologically normal individuals (without ET and with NC) are available from a study that used the Microarray Core BioCenter at the University of Texas Southwestern Medical Center (Stowe et al., unpublished results). In that study, in a subset of individuals aged 75 years and older (mean age = 78.5 ± 2.6 years), the mean ± standard deviation p-tau217 value was 0.44 ± 0.30 pg/mL, which is a value similar to the mean ± standard deviation value we report (0.3 ± 0.2 pg/mL) (personal communication with Dr. Dwight German, UT Southwestern Medical Center). Values for blood concentrations of p-tau181 and Aβ_42/40_ are available from three neurologically normal individuals (without ET and with NC) of similar age (median age = 74 years) to our ET cases, derived from the Microarray Core BioCenter at the University of Texas Southwestern Medical Center. The median values for p-tau181 (27.6 pg/mL) and Aβ_42/40_ (0.056 pg/mL) are similar to the median values we observed in our 20 ET cases with NC (24.6 pg/mL for p-tau181 and 0.06 pg/mL for Aβ_42/40_) (personal communication with Dr. Christian LoBue)_._ Hence, our ET cases with NC had values that are similar to those of individuals without ET who have NC.

We recognize several limitations. First, our sample size was limited to 40 participants among whom only 4 had dementia; hence, our conclusions should be tempered. Nonetheless, we were appropriately powered both to detect numerous significant associations between BBB concentrations and cognitive diagnosis as well as performance across numerous cognitive domains. We recognize, however, that several of the marginal associations we detected (e.g., p-tau217 concentration in ET dementia versus ET NC) could have reached statistical significance with a larger sample size. Second, replication of these results through the study of additional individuals is highly desirable. Third, the cross-sectional nature of the study did not allow us to assess the predictive value of biomarkers for cognitive decline over time. Future longitudinal analyses are therefore warranted. Finally, AD biomarkers and cognitive performance can be influenced by a variety of factors, and in these analyses, we considered age, sex, education, total number of prescription medications, cumulative illness, and depressive symptoms. However, we did not have access to measures of renal function.

The study also had numerous strengths. First, to our knowledge, this is the first time these biomarkers all have been assessed in a sample of ET cases as well as the first time biomarker concentrations have been correlated with cognitive performance in an ET population. Second, our ET cases were deeply phenotyped with a detailed neurological examination after which a senior movement disorders neurologist assigned ET diagnoses based on reliable and valid diagnostic criteria. Third, ET cases underwent an extensive neuropsychological assessment that allowed us to evaluate cognitive performance in detail across multiple cognitive domains. Fourth, for each participant, a cognitive diagnosis was assigned by a neuropsychologist and geriatric psychiatrist during a diagnostic consensus conference.

Although cognitive impairment is increasingly recognized in ET, there are currently no established fluid biomarkers for this condition. Albeit based on a small sample of cases, our findings suggest a potential role of BBB as markers for cognitive function in ET patients. Our results also suggest that cognitive decline in ET may be due to underlying neurodegenerative processes involving tau and perhaps Aβ pathology. Further research is needed to validate these biomarkers and explore their clinical utility in the context of diagnosing and predicting dementia in ET patients.
